# 1′-(1,3-Diphenyl-1*H*-pyrazol-4-yl)-1′′-(prop-2-en-1-yl)-2′,3′,5′,6′,7′,7a’-hexa­hydro-1′*H*-di­spiro­[1-benzo­pyran-3,2′-pyrrolizine-3′,3′′-indoline]-2′′,4-dione 0.75-hydrate

**DOI:** 10.1107/S160053681301773X

**Published:** 2013-07-03

**Authors:** G. Jagadeesan, D. Kathirvelan, J. Haribabu, B. S. R. Reddy, K. Sethusankar

**Affiliations:** a Department of Physics, Meenakshi College of Engineering, West K.K. Nagar, Chennai 600 078, India; b Industrial Chemistry Lab, Central Leather Research Institute, Adyar, Chennai 600 020, India; c Department of Physics, RKM Vivekananda College (Autonomous), Chennai 600 004, India

## Abstract

In the central aza-bi­cyclo­octane unit of the title compound, C_40_H_34_N_4_O_3_·0.75H_2_O, the peripheral pyrrolidine ring adopts an envelope conformation with the N atom deviating by 0.209 (2) Å, whereas the other pyrrolidine ring adopts a twisted conformation with the bridging N and C atoms deviating by −0.218 (2) and 0.236 (3) Å, respectively, from the rest of the ring. The pyrazole ring forms dihedral angles of 42.36 (7) and 24.07 (8)° with its C- and N-attached phenyl groups, respectively. The solvent water mol­ecule has a partial occupancy of 0.75. In the crystal, the water mol­ecules link the fused-ring mol­ecules into chains along the *b* axis *via* O—H⋯N and O—H⋯O hydrogen bonds. The crystal packing is further stabilized by C—H⋯π inter­actions involving a methyl­ene group of the pyran ring and the C-attached benzene ring on the pyrazole ring.

## Related literature

For the biological activity of pyrazole derivatives, see: Mahajan *et al.* (1991 ▶); Baraldi *et al.* (1998 ▶); Katayama & Oshiyama (1997 ▶); Chen & Li (1998 ▶). For a related structure, see: Jagadeesan *et al.* (2013 ▶). For puckering parameters, see: Cremer & Pople (1975 ▶).
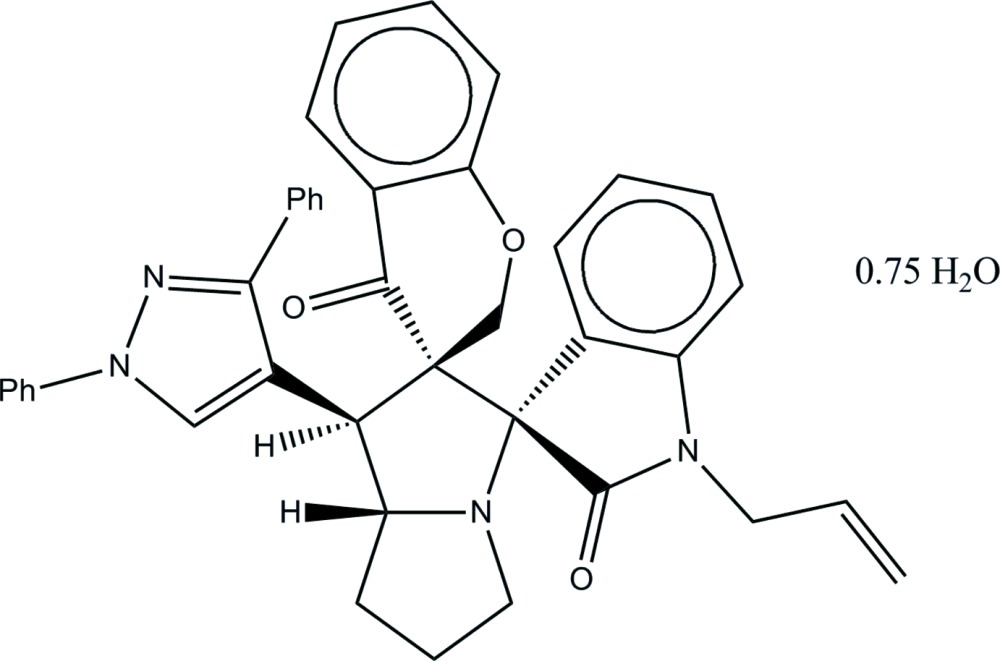


## Experimental

### 

#### Crystal data

C_40_H_34_N_4_O_3_·0.75H_2_O
*M* *_r_* = 632.22
Monoclinic, 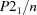

*a* = 11.451 (2) Å
*b* = 13.496 (2) Å
*c* = 20.815 (3) Å
β = 96.206 (9)°
*V* = 3198.0 (9) Å^3^
*Z* = 4
Mo *K*α radiation
μ = 0.09 mm^−1^
*T* = 296 K
0.30 × 0.25 × 0.20 mm

#### Data collection

Bruker Kappa APEXII CCD diffractometer
Absorption correction: multi-scan (*SADABS*; Bruker, 2008 ▶) *T*_min_ = 0.975, *T*_max_ = 0.983
32619 measured reflections
6870 independent reflections
4468 reflections with *I* > 2σ(*I*)
*R*_int_ = 0.041

#### Refinement

*R*[*F* ^2^ > 2σ(*F* ^2^)] = 0.051
*wR*(*F* ^2^) = 0.153
*S* = 1.02
6870 reflections
439 parameters
3 restraints
H atoms treated by a mixture of independent and constrained refinement
Δρ_max_ = 0.53 e Å^−3^
Δρ_min_ = −0.25 e Å^−3^

### 

Data collection: *APEX2* (Bruker, 2008 ▶); cell refinement: *SAINT* (Bruker, 2008 ▶); data reduction: *SAINT*; program(s) used to solve structure: *SHELXS97* (Sheldrick, 2008 ▶); program(s) used to refine structure: *SHELXL97* (Sheldrick, 2008 ▶); molecular graphics: *ORTEP-3 for Windows* (Farrugia, 2012 ▶); software used to prepare material for publication: *SHELXL97* and *PLATON* (Spek, 2009 ▶).

## Supplementary Material

Crystal structure: contains datablock(s) global, I. DOI: 10.1107/S160053681301773X/ld2105sup1.cif

Structure factors: contains datablock(s) I. DOI: 10.1107/S160053681301773X/ld2105Isup2.hkl

Additional supplementary materials:  crystallographic information; 3D view; checkCIF report

## Figures and Tables

**Table 1:** Hydrogen-bond geometry (Å, °) *Cg*1 is the centroid of the C1–C6 ring.

*D*—H⋯*A*	*D*—H	H⋯*A*	*D*⋯*A*	*D*—H⋯*A*
O4*W*—H1*W*⋯N3^i^	0.91 (2)	2.02 (3)	2.892 (4)	161 (4)
O4*W*—H2*W*⋯O2^ii^	0.90 (1)	1.96 (1)	2.841 (3)	165 (3)
C40—H40*A*⋯*Cg*1^iii^	0.97	2.78	3.540 (3)	136

Symmetry codes: (i) 
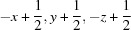
; (ii) 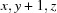; (iii) 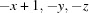.

**Fig. 1.**
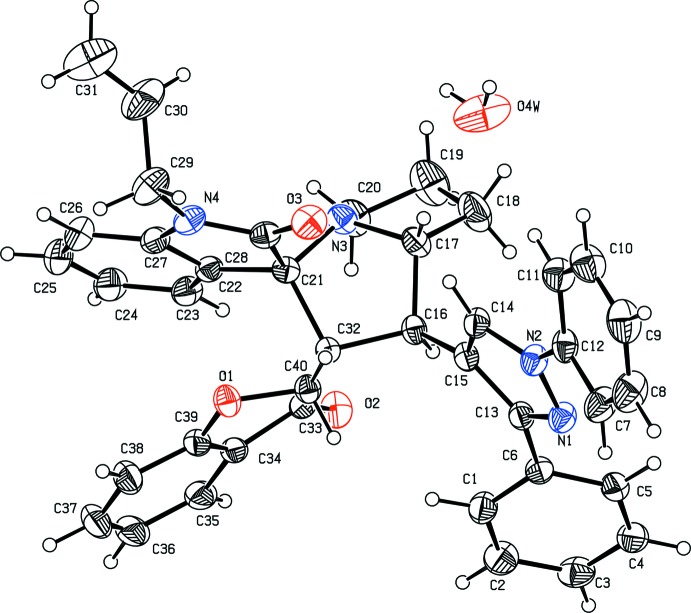
The molecular structure of the title compound showing 30% probability displacement ellipsoids.

**Fig. 2.**
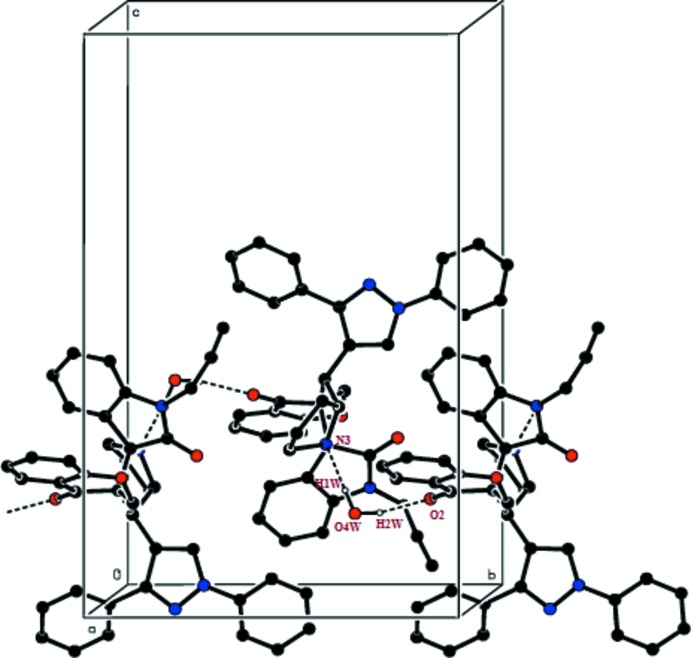
The crystal packing of the title compound, viewed down the a axis showing intermolecular O—H···N and O—H···O hydrogen bonds (dashed lines). The H–atoms not involved in hydrogen bonds have been excluded for clarity.

**Fig. 3.**
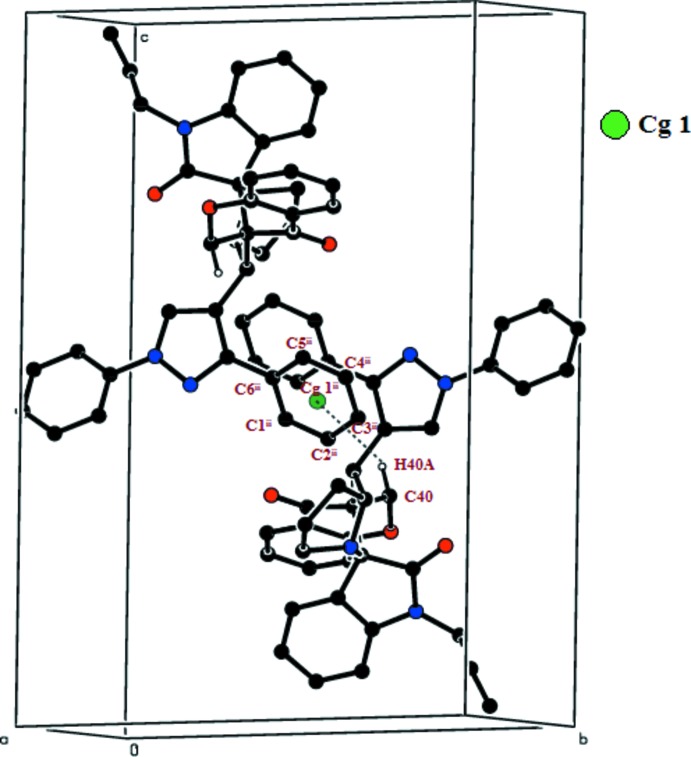
The crystal packing arrangement of the title compound viewed down the a axis showing intermolecular C—H···π hydrogen bonds (dashed lines). The isolated water molecule and H–atoms not involved in hydrogen bonds have been excluded for clarity.

## supplementary crystallographic information

### Comment

Pyrazole derivatives in general are well known nitrogen containing heterocyclic compounds and have been the subject of enormous research due to their importance in various applications and their widespread potential biological and pharmacological activities such as antimicrobial (Mahajan *et al.*, 1991), antiviral (Baraldi *et al.*, 1998), antitumor (Katayama & Oshiyama, 1997) and antifungal activities (Chen & Li, 1998).

The molecular structure of the title compound C_40_H_34_N_4_O_3_.0.75H_2_O, is shown in Fig. 1. The mean planes of the phenyl rings (C1–C6) and (C7–C12) form a dihedral angle of 62.08 (7)° between them. The mean plane of the pyrazole ring (C13/C14/C15/N1/N2) forms dihedral angles of 42.36 (7)° and 24.07 (8)° with the mean planes of the two phenyl rings (C1–C6) and (C7–C12), respectively. The mean plane of the pyrrolizine ring (C16–C21/C32/N3) forms dihedral angles of 78.04 (7)° and 74.97 (6)° with the mean planes of the chromene ring (C32–C40/O1) and the indole ring (C21–C28/N4), respectively. The solvent water molecule is partially occupied, with a refined occupancy of 0.75.

The sum of angles around the N2 atom (356 °) indicates *sp*^2^ hybridization, whereas that around N3 atom (336.7 °) indicates *sp*^3^ hybridization. The pyrrolidine ring (C16/C17/C21/C32/N3) adopts an *envelope* conformation on N3, with puckering parameters (Cremer & Pople, 1975) of q_2_ = 0.332 (2) Å and φ_2_= 187.4 (4)°. Also, the atom N3 deviates from the mean planes of the remaining ring atoms by -0.208 (19) Å. The other pyrrolidine ring (C17–C20/N3) adopts a *twisted* conformation on N3 and C20, with puckering parameters of q_2_ = 0.380 (2) Å and φ_2_= 155.3 (4)°. Also, the atoms N3 and C20 deviate from the mean planes of the remaining ring atoms by -0.218 (2) Å and 0.236 (3) Å, respectively. The title compound exhibits structural similarities with an already reported related structure (Jagadeesan *et al.*, 2013).

In the crystal packing, the molecules are linked to the water molecules *via* intermolecular O4W–H1W···N3^i^ and O4W–H2W···O2^ii^ interactions is view down the *a* axis. The crystal packing is further stabilized by C40–H40A···*Cg*1^iii^ hydrogen bond interaction, where *Cg*1 is the center of gravity of (C1–C6) ring (Table 1). The symmetry codes: (i) -*x* + 1/2, *y* + 1/2, -*z* + 1/2, (ii) *x*, *y* + 1, *z*, (iii) -*x* + 1, -*y*, -*z*. The packing view of the title compound is shown in Fig. 2 and Fig. 3.

### Experimental

A mixture of allyl isatin (1.05 mmol), sarcosine (1.1 mmol) and dipolarophile (1.0 mmol) in ethanol was refluxed for 85 minute and cooled to room temperature. Then the mixture was poured into a beaker containing crushed ice and the solid formed in the mixture was filtered, dried and recrystallized from ethanol to obtain the pure product in good yield 89%.

### Refinement

Hydrogen atoms were placed in calculated positions with C–H = 0.93 to 0.98 Å refined in the riding model with fixed isotropic displacement parameters: *U*_iso_(H) = 1.2 *U*_eq_(C). The water H atoms were located in a difference map and refined with distance restraints of O–H = 0.90 (1) Å. The solvent water molecule is partially occupied, with an occupancy factor of 0.75.

### Crystal data

**Table:**

C_40_H_34_N_4_O_3_·0.75H_2_O	*F*(000) = 1334
*M**_r_* = 632.22	*D*_x_ = 1.313 Mg m^−^^3^
Monoclinic, *P*2_1_/*n*	Mo *K*α radiation, λ = 0.71073 Å
Hall symbol: -P 2yn	Cell parameters from 6870 reflections
*a* = 11.451 (2) Å	θ = 2.0–26.9°
*b* = 13.496 (2) Å	µ = 0.09 mm^−^^1^
*c* = 20.815 (3) Å	*T* = 296 K
β = 96.206 (9)°	Block, colourless
*V* = 3198.0 (9) Å^3^	0.30 × 0.25 × 0.20 mm
*Z* = 4	

### Data collection

**Table:**

Bruker Kappa APEXII CCD diffractometer	6870 independent reflections
Radiation source: fine-focus sealed tube	4468 reflections with *I* > 2σ(*I*)
Graphite monochromator	*R*_int_ = 0.041
ω scans	θ_max_ = 26.9°, θ_min_ = 2.0°
Absorption correction: multi-scan (*SADABS*; Bruker, 2008)	*h* = −14→14
*T*_min_ = 0.975, *T*_max_ = 0.983	*k* = −17→16
32619 measured reflections	*l* = −26→26

### Refinement

**Table:**

Refinement on *F*^2^	Primary atom site location: structure-invariant direct methods
Least-squares matrix: full	Secondary atom site location: difference Fourier map
*R*[*F*^2^ > 2σ(*F*^2^)] = 0.051	Hydrogen site location: inferred from neighbouring sites
*wR*(*F*^2^) = 0.153	H atoms treated by a mixture of independent and constrained refinement
*S* = 1.02	*w* = 1/[σ^2^(*F*_o_^2^) + (0.0677*P*)^2^ + 1.1541*P*] where *P* = (*F*_o_^2^ + 2*F*_c_^2^)/3
6870 reflections	(Δ/σ)_max_ < 0.001
439 parameters	Δρ_max_ = 0.53 e Å^−^^3^
3 restraints	Δρ_min_ = −0.25 e Å^−^^3^

### Special details

**Table:**

Geometry. All e.s.d.'s (except the e.s.d. in the dihedral angle between two l.s. planes) are estimated using the full covariance matrix. The cell e.s.d.'s are taken into account individually in the estimation of e.s.d.'s in distances, angles and torsion angles; correlations between e.s.d.'s in cell parameters are only used when they are defined by crystal symmetry. An approximate (isotropic) treatment of cell e.s.d.'s is used for estimating e.s.d.'s involving l.s. planes.
Refinement. Refinement of *F*^2^ against ALL reflections. The weighted *R*-factor *wR* and goodness of fit *S* are based on *F*^2^, conventional *R*-factors *R* are based on *F*, with *F* set to zero for negative *F*^2^. The threshold expression of *F*^2^ > σ(*F*^2^) is used only for calculating *R*-factors(gt) *etc*. and is not relevant to the choice of reflections for refinement. *R*-factors based on *F*^2^ are statistically about twice as large as those based on *F*, and *R*- factors based on ALL data will be even larger.

### Fractional atomic coordinates and isotropic or equivalent isotropic displacement parameters (Å^2^)

**Table:**

	*x*	*y*	*z*	*U*_iso_*/*U*_eq_	Occ. (<1)
C1	0.3646 (2)	−0.09434 (16)	0.00940 (10)	0.0520 (5)	
H1	0.4169	−0.0873	0.0465	0.062*	
C2	0.3490 (2)	−0.18622 (17)	−0.01924 (12)	0.0618 (6)	
H2	0.3895	−0.2409	−0.0010	0.074*	
C3	0.2736 (2)	−0.19691 (18)	−0.07481 (12)	0.0590 (6)	
H3	0.2620	−0.2590	−0.0938	0.071*	
C4	0.21576 (19)	−0.11638 (18)	−0.10203 (11)	0.0551 (6)	
H4	0.1664	−0.1234	−0.1403	0.066*	
C5	0.23008 (18)	−0.02496 (16)	−0.07328 (10)	0.0476 (5)	
H5	0.1900	0.0294	−0.0922	0.057*	
C6	0.30383 (17)	−0.01291 (14)	−0.01625 (9)	0.0407 (5)	
C7	0.3903 (2)	0.35239 (18)	−0.06490 (11)	0.0619 (6)	
H7	0.4125	0.2978	−0.0880	0.074*	
C8	0.4026 (3)	0.4471 (2)	−0.08857 (13)	0.0741 (8)	
H8	0.4335	0.4562	−0.1277	0.089*	
C9	0.3697 (3)	0.52715 (19)	−0.05491 (14)	0.0726 (8)	
H9	0.3775	0.5906	−0.0713	0.087*	
C10	0.3252 (2)	0.51435 (18)	0.00313 (14)	0.0676 (7)	
H10	0.3040	0.5693	0.0262	0.081*	
C11	0.3118 (2)	0.42039 (16)	0.02735 (12)	0.0565 (6)	
H11	0.2807	0.4117	0.0664	0.068*	
C12	0.34474 (19)	0.33968 (15)	−0.00683 (10)	0.0462 (5)	
N2	0.33424 (15)	0.24231 (12)	0.01741 (8)	0.0442 (4)	
C14	0.33160 (18)	0.21340 (15)	0.07935 (9)	0.0432 (5)	
H14	0.3362	0.2548	0.1153	0.052*	
C15	0.32098 (17)	0.11219 (14)	0.07987 (9)	0.0390 (4)	
C16	0.30271 (16)	0.04839 (14)	0.13713 (9)	0.0378 (4)	
H16	0.2682	−0.0142	0.1205	0.045*	
C17	0.21513 (18)	0.09593 (17)	0.17936 (10)	0.0475 (5)	
H17	0.2233	0.1682	0.1789	0.057*	
C18	0.0875 (2)	0.0674 (2)	0.16208 (14)	0.0777 (8)	
H18A	0.0379	0.1259	0.1588	0.093*	
H18B	0.0774	0.0323	0.1212	0.093*	
C19	0.0572 (2)	0.0020 (2)	0.21576 (17)	0.0856 (9)	
H19A	0.0121	−0.0548	0.1988	0.103*	
H19B	0.0116	0.0381	0.2447	0.103*	
C20	0.1725 (2)	−0.03022 (19)	0.25043 (13)	0.0641 (7)	
H20A	0.1651	−0.0465	0.2952	0.077*	
H20B	0.2041	−0.0869	0.2295	0.077*	
C21	0.37282 (17)	0.05038 (14)	0.25424 (9)	0.0373 (4)	
C22	0.41957 (17)	−0.01102 (15)	0.31226 (9)	0.0401 (4)	
C23	0.4085 (2)	−0.10970 (16)	0.32683 (10)	0.0485 (5)	
H23	0.3644	−0.1518	0.2983	0.058*	
C24	0.4642 (2)	−0.14571 (18)	0.38482 (11)	0.0604 (6)	
H24	0.4570	−0.2122	0.3953	0.072*	
C25	0.5296 (2)	−0.0836 (2)	0.42650 (11)	0.0663 (7)	
H25	0.5675	−0.1092	0.4647	0.080*	
C26	0.5407 (2)	0.0153 (2)	0.41352 (11)	0.0609 (6)	
H26	0.5843	0.0572	0.4424	0.073*	
C27	0.48488 (18)	0.05012 (16)	0.35618 (9)	0.0441 (5)	
C28	0.42046 (18)	0.15474 (15)	0.27428 (10)	0.0432 (5)	
C29	0.5406 (2)	0.23204 (18)	0.36684 (12)	0.0657 (7)	
H29A	0.5566	0.2825	0.3358	0.079*	
H29B	0.6149	0.2112	0.3896	0.079*	
C30	0.4627 (4)	0.2760 (2)	0.41537 (14)	0.0914 (10)	
H30	0.3821	0.2748	0.4034	0.110*	
C31	0.4951 (4)	0.3122 (3)	0.46756 (18)	0.1216 (14)	
H31A	0.5748	0.3153	0.4819	0.146*	
H31B	0.4402	0.3369	0.4933	0.146*	
C32	0.41258 (16)	0.02229 (13)	0.18492 (8)	0.0332 (4)	
C33	0.44132 (18)	−0.08767 (14)	0.18398 (9)	0.0384 (4)	
C34	0.56279 (18)	−0.11499 (15)	0.20664 (9)	0.0422 (5)	
C35	0.5953 (2)	−0.21383 (18)	0.21540 (10)	0.0574 (6)	
H35	0.5416	−0.2637	0.2027	0.069*	
C36	0.7059 (3)	−0.2382 (2)	0.24262 (12)	0.0723 (8)	
H36	0.7278	−0.3043	0.2478	0.087*	
C37	0.7845 (3)	−0.1643 (3)	0.26227 (13)	0.0760 (8)	
H37	0.8590	−0.1811	0.2813	0.091*	
C38	0.7549 (2)	−0.0671 (2)	0.25432 (12)	0.0619 (6)	
H38	0.8088	−0.0179	0.2681	0.074*	
C39	0.64463 (18)	−0.04197 (16)	0.22576 (9)	0.0445 (5)	
C40	0.52334 (16)	0.07518 (15)	0.16948 (9)	0.0394 (4)	
H40A	0.5435	0.0533	0.1277	0.047*	
H40B	0.5088	0.1460	0.1670	0.047*	
O4W	0.3354 (3)	0.6451 (2)	0.14051 (16)	0.1037 (10)	0.75
N1	0.32593 (16)	0.16460 (12)	−0.02365 (8)	0.0460 (4)	
C13	0.31812 (17)	0.08557 (14)	0.01392 (9)	0.0398 (4)	
N3	0.24531 (15)	0.05827 (13)	0.24518 (8)	0.0457 (4)	
N4	0.48377 (16)	0.14756 (13)	0.33290 (8)	0.0478 (4)	
O1	0.61974 (11)	0.05582 (11)	0.21763 (7)	0.0460 (4)	
O2	0.36699 (14)	−0.14969 (10)	0.16740 (7)	0.0555 (4)	
O3	0.40355 (15)	0.23089 (10)	0.24343 (7)	0.0570 (4)	
H2W	0.337 (3)	0.7119 (8)	0.1421 (15)	0.086*	0.75
H1W	0.328 (4)	0.618 (2)	0.1800 (9)	0.086*	0.75

### Atomic displacement parameters (Å^2^)

**Table:**

	*U*^11^	*U*^22^	*U*^33^	*U*^12^	*U*^13^	*U*^23^
C1	0.0632 (14)	0.0459 (13)	0.0450 (11)	0.0053 (11)	−0.0024 (10)	−0.0013 (10)
C2	0.0813 (18)	0.0418 (13)	0.0620 (14)	0.0087 (12)	0.0054 (13)	0.0002 (11)
C3	0.0612 (15)	0.0484 (14)	0.0686 (15)	−0.0075 (12)	0.0120 (12)	−0.0180 (12)
C4	0.0430 (13)	0.0641 (15)	0.0570 (13)	−0.0029 (11)	0.0001 (10)	−0.0183 (12)
C5	0.0421 (12)	0.0519 (13)	0.0478 (11)	0.0041 (10)	0.0008 (9)	−0.0050 (10)
C6	0.0422 (11)	0.0390 (11)	0.0410 (10)	−0.0008 (9)	0.0052 (8)	−0.0009 (8)
C7	0.0895 (19)	0.0483 (14)	0.0472 (12)	0.0022 (13)	0.0042 (12)	0.0065 (10)
C8	0.104 (2)	0.0608 (17)	0.0568 (14)	−0.0025 (15)	0.0055 (14)	0.0200 (13)
C9	0.087 (2)	0.0464 (15)	0.0810 (19)	−0.0020 (13)	−0.0072 (15)	0.0213 (14)
C10	0.0740 (18)	0.0412 (13)	0.0847 (19)	0.0039 (12)	−0.0038 (14)	0.0015 (13)
C11	0.0641 (15)	0.0432 (13)	0.0609 (14)	0.0014 (11)	0.0015 (11)	0.0020 (11)
C12	0.0523 (13)	0.0381 (11)	0.0453 (11)	−0.0017 (9)	−0.0074 (9)	0.0067 (9)
N2	0.0552 (11)	0.0351 (9)	0.0409 (9)	−0.0004 (8)	−0.0020 (8)	0.0023 (7)
C14	0.0498 (12)	0.0396 (11)	0.0384 (10)	0.0017 (9)	−0.0031 (9)	0.0009 (9)
C15	0.0370 (11)	0.0371 (11)	0.0413 (10)	0.0017 (8)	−0.0031 (8)	0.0019 (8)
C16	0.0361 (10)	0.0343 (10)	0.0417 (10)	−0.0035 (8)	−0.0025 (8)	0.0014 (8)
C17	0.0373 (11)	0.0508 (13)	0.0547 (12)	0.0059 (9)	0.0069 (9)	0.0107 (10)
C18	0.0375 (13)	0.109 (2)	0.0862 (19)	0.0051 (14)	0.0043 (13)	0.0211 (17)
C19	0.0485 (16)	0.091 (2)	0.116 (2)	−0.0173 (15)	0.0034 (15)	0.0197 (18)
C20	0.0529 (15)	0.0649 (16)	0.0758 (16)	−0.0104 (12)	0.0124 (12)	0.0119 (13)
C21	0.0393 (11)	0.0324 (10)	0.0406 (10)	−0.0005 (8)	0.0062 (8)	0.0013 (8)
C22	0.0434 (11)	0.0420 (11)	0.0358 (9)	0.0031 (9)	0.0088 (8)	0.0012 (8)
C23	0.0607 (14)	0.0423 (12)	0.0436 (11)	0.0033 (10)	0.0109 (10)	0.0054 (9)
C24	0.0782 (17)	0.0538 (14)	0.0506 (13)	0.0118 (12)	0.0142 (12)	0.0142 (11)
C25	0.0786 (18)	0.0758 (18)	0.0431 (12)	0.0169 (14)	0.0001 (12)	0.0134 (12)
C26	0.0662 (16)	0.0733 (17)	0.0416 (12)	0.0021 (13)	−0.0016 (11)	−0.0032 (11)
C27	0.0473 (12)	0.0467 (12)	0.0392 (10)	0.0014 (9)	0.0082 (9)	−0.0041 (9)
C28	0.0486 (12)	0.0375 (11)	0.0446 (11)	0.0000 (9)	0.0098 (9)	−0.0037 (9)
C29	0.0843 (19)	0.0569 (15)	0.0557 (14)	−0.0255 (13)	0.0060 (13)	−0.0129 (12)
C30	0.150 (3)	0.0558 (17)	0.0662 (17)	−0.0111 (18)	0.0020 (19)	−0.0303 (14)
C31	0.150 (4)	0.121 (3)	0.101 (3)	−0.014 (3)	0.042 (3)	−0.026 (2)
C32	0.0335 (10)	0.0302 (9)	0.0354 (9)	−0.0008 (7)	0.0021 (7)	0.0011 (7)
C33	0.0477 (12)	0.0341 (10)	0.0335 (9)	0.0002 (9)	0.0051 (8)	0.0001 (8)
C34	0.0488 (12)	0.0438 (11)	0.0347 (9)	0.0123 (9)	0.0079 (8)	0.0032 (8)
C35	0.0748 (16)	0.0505 (13)	0.0478 (12)	0.0216 (12)	0.0103 (11)	0.0027 (10)
C36	0.085 (2)	0.0721 (18)	0.0613 (15)	0.0433 (16)	0.0147 (14)	0.0145 (13)
C37	0.0571 (16)	0.106 (2)	0.0645 (16)	0.0366 (17)	0.0062 (13)	0.0196 (16)
C38	0.0425 (13)	0.0850 (19)	0.0575 (13)	0.0096 (12)	0.0022 (10)	0.0089 (13)
C39	0.0391 (11)	0.0561 (13)	0.0389 (10)	0.0074 (10)	0.0075 (8)	0.0036 (9)
C40	0.0358 (10)	0.0412 (11)	0.0407 (10)	−0.0019 (8)	0.0022 (8)	0.0032 (8)
O4W	0.156 (3)	0.0594 (16)	0.107 (2)	−0.0196 (18)	0.066 (2)	−0.0095 (15)
N1	0.0571 (11)	0.0391 (10)	0.0407 (9)	0.0014 (8)	−0.0007 (8)	0.0003 (8)
C13	0.0395 (11)	0.0384 (11)	0.0401 (10)	0.0033 (8)	−0.0015 (8)	0.0011 (8)
N3	0.0400 (9)	0.0472 (10)	0.0509 (10)	−0.0007 (8)	0.0100 (7)	0.0076 (8)
N4	0.0564 (11)	0.0432 (10)	0.0441 (9)	−0.0071 (8)	0.0068 (8)	−0.0091 (8)
O1	0.0355 (7)	0.0502 (9)	0.0510 (8)	−0.0042 (6)	−0.0018 (6)	0.0035 (7)
O2	0.0629 (10)	0.0363 (8)	0.0647 (10)	−0.0066 (7)	−0.0055 (8)	0.0004 (7)
O3	0.0769 (11)	0.0326 (8)	0.0617 (9)	−0.0020 (7)	0.0081 (8)	0.0016 (7)

### Geometric parameters (Å, º)

**Table:**

C1—C6	1.377 (3)	C21—C22	1.514 (3)
C1—C2	1.379 (3)	C21—C28	1.551 (3)
C1—H1	0.9300	C21—C32	1.605 (3)
C2—C3	1.374 (3)	C22—C23	1.375 (3)
C2—H2	0.9300	C22—C27	1.388 (3)
C3—C4	1.364 (3)	C23—C24	1.390 (3)
C3—H3	0.9300	C23—H23	0.9300
C4—C5	1.373 (3)	C24—C25	1.370 (4)
C4—H4	0.9300	C24—H24	0.9300
C5—C6	1.390 (3)	C25—C26	1.370 (4)
C5—H5	0.9300	C25—H25	0.9300
C6—C13	1.472 (3)	C26—C27	1.374 (3)
C7—C12	1.378 (3)	C26—H26	0.9300
C7—C8	1.382 (3)	C27—N4	1.401 (3)
C7—H7	0.9300	C28—O3	1.216 (2)
C8—C9	1.363 (4)	C28—N4	1.354 (3)
C8—H8	0.9300	C29—N4	1.457 (3)
C9—C10	1.372 (4)	C29—C30	1.537 (4)
C9—H9	0.9300	C29—H29A	0.9700
C10—C11	1.379 (3)	C29—H29B	0.9700
C10—H10	0.9300	C30—C31	1.212 (4)
C11—C12	1.376 (3)	C30—H30	0.9300
C11—H11	0.9300	C31—H31A	0.9300
C12—N2	1.417 (3)	C31—H31B	0.9300
N2—N1	1.350 (2)	C32—C40	1.520 (3)
N2—C14	1.351 (2)	C32—C33	1.521 (3)
C14—C15	1.371 (3)	C33—O2	1.217 (2)
C14—H14	0.9300	C33—C34	1.467 (3)
C15—C13	1.416 (3)	C34—C39	1.388 (3)
C15—C16	1.503 (3)	C34—C35	1.392 (3)
C16—C17	1.543 (3)	C35—C36	1.371 (4)
C16—C32	1.558 (3)	C35—H35	0.9300
C16—H16	0.9800	C36—C37	1.374 (4)
C17—N3	1.467 (3)	C36—H36	0.9300
C17—C18	1.517 (3)	C37—C38	1.361 (4)
C17—H17	0.9800	C37—H37	0.9300
C18—C19	1.493 (4)	C38—C39	1.379 (3)
C18—H18A	0.9700	C38—H38	0.9300
C18—H18B	0.9700	C39—O1	1.357 (3)
C19—C20	1.499 (4)	C40—O1	1.433 (2)
C19—H19A	0.9700	C40—H40A	0.9700
C19—H19B	0.9700	C40—H40B	0.9700
C20—N3	1.468 (3)	O4W—H2W	0.902 (10)
C20—H20A	0.9700	O4W—H1W	0.912 (10)
C20—H20B	0.9700	N1—C13	1.331 (2)
C21—N3	1.456 (3)		
			
C6—C1—C2	120.9 (2)	C28—C21—C32	109.30 (14)
C6—C1—H1	119.6	C23—C22—C27	119.11 (19)
C2—C1—H1	119.6	C23—C22—C21	132.30 (19)
C3—C2—C1	119.9 (2)	C27—C22—C21	108.60 (17)
C3—C2—H2	120.0	C22—C23—C24	119.0 (2)
C1—C2—H2	120.0	C22—C23—H23	120.5
C4—C3—C2	119.9 (2)	C24—C23—H23	120.5
C4—C3—H3	120.0	C25—C24—C23	120.3 (2)
C2—C3—H3	120.0	C25—C24—H24	119.9
C3—C4—C5	120.3 (2)	C23—C24—H24	119.9
C3—C4—H4	119.8	C24—C25—C26	121.8 (2)
C5—C4—H4	119.8	C24—C25—H25	119.1
C4—C5—C6	120.7 (2)	C26—C25—H25	119.1
C4—C5—H5	119.7	C25—C26—C27	117.4 (2)
C6—C5—H5	119.7	C25—C26—H26	121.3
C1—C6—C5	118.22 (19)	C27—C26—H26	121.3
C1—C6—C13	121.79 (18)	C26—C27—C22	122.4 (2)
C5—C6—C13	119.96 (18)	C26—C27—N4	127.4 (2)
C12—C7—C8	119.4 (2)	C22—C27—N4	110.24 (17)
C12—C7—H7	120.3	O3—C28—N4	125.15 (19)
C8—C7—H7	120.3	O3—C28—C21	126.43 (18)
C9—C8—C7	120.3 (3)	N4—C28—C21	108.42 (17)
C9—C8—H8	119.8	N4—C29—C30	111.1 (2)
C7—C8—H8	119.8	N4—C29—H29A	109.4
C8—C9—C10	120.2 (2)	C30—C29—H29A	109.4
C8—C9—H9	119.9	N4—C29—H29B	109.4
C10—C9—H9	119.9	C30—C29—H29B	109.4
C9—C10—C11	120.3 (3)	H29A—C29—H29B	108.0
C9—C10—H10	119.9	C31—C30—C29	127.0 (4)
C11—C10—H10	119.9	C31—C30—H30	116.5
C12—C11—C10	119.4 (2)	C29—C30—H30	116.5
C12—C11—H11	120.3	C30—C31—H31A	120.0
C10—C11—H11	120.3	C30—C31—H31B	120.0
C11—C12—C7	120.4 (2)	H31A—C31—H31B	120.0
C11—C12—N2	120.8 (2)	C40—C32—C33	105.60 (15)
C7—C12—N2	118.8 (2)	C40—C32—C16	113.19 (15)
N1—N2—C14	111.88 (16)	C33—C32—C16	111.93 (15)
N1—N2—C12	119.87 (16)	C40—C32—C21	113.81 (15)
C14—N2—C12	128.25 (17)	C33—C32—C21	108.96 (14)
N2—C14—C15	107.84 (17)	C16—C32—C21	103.45 (14)
N2—C14—H14	126.1	O2—C33—C34	121.89 (18)
C15—C14—H14	126.1	O2—C33—C32	121.95 (18)
C14—C15—C13	103.76 (17)	C34—C33—C32	116.12 (17)
C14—C15—C16	126.64 (18)	C39—C34—C35	118.7 (2)
C13—C15—C16	129.25 (17)	C39—C34—C33	120.07 (18)
C15—C16—C17	111.61 (16)	C35—C34—C33	120.9 (2)
C15—C16—C32	117.64 (15)	C36—C35—C34	120.4 (3)
C17—C16—C32	105.08 (15)	C36—C35—H35	119.8
C15—C16—H16	107.3	C34—C35—H35	119.8
C17—C16—H16	107.3	C35—C36—C37	119.7 (2)
C32—C16—H16	107.3	C35—C36—H36	120.2
N3—C17—C18	104.90 (18)	C37—C36—H36	120.2
N3—C17—C16	106.62 (16)	C38—C37—C36	121.1 (2)
C18—C17—C16	115.4 (2)	C38—C37—H37	119.4
N3—C17—H17	109.9	C36—C37—H37	119.4
C18—C17—H17	109.9	C37—C38—C39	119.6 (3)
C16—C17—H17	109.9	C37—C38—H38	120.2
C19—C18—C17	105.5 (2)	C39—C38—H38	120.2
C19—C18—H18A	110.6	O1—C39—C38	117.6 (2)
C17—C18—H18A	110.6	O1—C39—C34	121.90 (18)
C19—C18—H18B	110.6	C38—C39—C34	120.5 (2)
C17—C18—H18B	110.6	O1—C40—C32	111.35 (15)
H18A—C18—H18B	108.8	O1—C40—H40A	109.4
C18—C19—C20	105.6 (2)	C32—C40—H40A	109.4
C18—C19—H19A	110.6	O1—C40—H40B	109.4
C20—C19—H19A	110.6	C32—C40—H40B	109.4
C18—C19—H19B	110.6	H40A—C40—H40B	108.0
C20—C19—H19B	110.6	H2W—O4W—H1W	111.8 (16)
H19A—C19—H19B	108.8	C13—N1—N2	104.78 (16)
N3—C20—C19	101.9 (2)	N1—C13—C15	111.74 (17)
N3—C20—H20A	111.4	N1—C13—C6	118.97 (17)
C19—C20—H20A	111.4	C15—C13—C6	129.25 (17)
N3—C20—H20B	111.4	C21—N3—C17	106.14 (15)
C19—C20—H20B	111.4	C21—N3—C20	120.07 (17)
H20A—C20—H20B	109.3	C17—N3—C20	105.84 (18)
N3—C21—C22	113.99 (15)	C28—N4—C27	111.27 (16)
N3—C21—C28	106.68 (15)	C28—N4—C29	123.27 (19)
C22—C21—C28	101.45 (15)	C27—N4—C29	125.44 (18)
N3—C21—C32	106.20 (15)	C39—O1—C40	113.59 (15)
C22—C21—C32	118.54 (15)		
			
C6—C1—C2—C3	1.2 (4)	C28—C21—C32—C40	25.4 (2)
C1—C2—C3—C4	1.0 (4)	N3—C21—C32—C33	−102.37 (17)
C2—C3—C4—C5	−1.7 (4)	C22—C21—C32—C33	27.4 (2)
C3—C4—C5—C6	0.2 (3)	C28—C21—C32—C33	142.89 (16)
C2—C1—C6—C5	−2.7 (3)	N3—C21—C32—C16	16.86 (18)
C2—C1—C6—C13	179.3 (2)	C22—C21—C32—C16	146.65 (16)
C4—C5—C6—C1	2.0 (3)	C28—C21—C32—C16	−97.88 (17)
C4—C5—C6—C13	−179.98 (19)	C40—C32—C33—O2	−148.26 (18)
C12—C7—C8—C9	−0.2 (4)	C16—C32—C33—O2	−24.7 (3)
C7—C8—C9—C10	0.7 (4)	C21—C32—C33—O2	89.1 (2)
C8—C9—C10—C11	−1.0 (4)	C40—C32—C33—C34	34.1 (2)
C9—C10—C11—C12	0.8 (4)	C16—C32—C33—C34	157.65 (15)
C10—C11—C12—C7	−0.3 (4)	C21—C32—C33—C34	−88.56 (19)
C10—C11—C12—N2	178.7 (2)	O2—C33—C34—C39	−179.32 (19)
C8—C7—C12—C11	0.0 (4)	C32—C33—C34—C39	−1.6 (3)
C8—C7—C12—N2	−179.0 (2)	O2—C33—C34—C35	−5.7 (3)
C11—C12—N2—N1	156.5 (2)	C32—C33—C34—C35	172.01 (18)
C7—C12—N2—N1	−24.5 (3)	C39—C34—C35—C36	0.2 (3)
C11—C12—N2—C14	−23.8 (3)	C33—C34—C35—C36	−173.5 (2)
C7—C12—N2—C14	155.2 (2)	C34—C35—C36—C37	1.0 (4)
N1—N2—C14—C15	−0.1 (2)	C35—C36—C37—C38	−1.0 (4)
C12—N2—C14—C15	−179.7 (2)	C36—C37—C38—C39	−0.4 (4)
N2—C14—C15—C13	0.2 (2)	C37—C38—C39—O1	−178.7 (2)
N2—C14—C15—C16	−173.55 (18)	C37—C38—C39—C34	1.7 (3)
C14—C15—C16—C17	40.7 (3)	C35—C34—C39—O1	178.81 (18)
C13—C15—C16—C17	−131.4 (2)	C33—C34—C39—O1	−7.4 (3)
C14—C15—C16—C32	−80.9 (3)	C35—C34—C39—C38	−1.6 (3)
C13—C15—C16—C32	107.0 (2)	C33—C34—C39—C38	172.20 (19)
C15—C16—C17—N3	−153.12 (16)	C33—C32—C40—O1	−62.21 (19)
C32—C16—C17—N3	−24.6 (2)	C16—C32—C40—O1	175.01 (15)
C15—C16—C17—C18	90.9 (2)	C21—C32—C40—O1	57.3 (2)
C32—C16—C17—C18	−140.57 (19)	C14—N2—N1—C13	−0.1 (2)
N3—C17—C18—C19	−8.0 (3)	C12—N2—N1—C13	179.63 (18)
C16—C17—C18—C19	109.0 (3)	N2—N1—C13—C15	0.2 (2)
C17—C18—C19—C20	−16.8 (3)	N2—N1—C13—C6	178.01 (17)
C18—C19—C20—N3	35.1 (3)	C14—C15—C13—N1	−0.2 (2)
N3—C21—C22—C23	64.7 (3)	C16—C15—C13—N1	173.26 (19)
C28—C21—C22—C23	178.9 (2)	C14—C15—C13—C6	−177.8 (2)
C32—C21—C22—C23	−61.5 (3)	C16—C15—C13—C6	−4.3 (3)
N3—C21—C22—C27	−115.54 (19)	C1—C6—C13—N1	137.5 (2)
C28—C21—C22—C27	−1.3 (2)	C5—C6—C13—N1	−40.5 (3)
C32—C21—C22—C27	118.32 (18)	C1—C6—C13—C15	−45.1 (3)
C27—C22—C23—C24	−0.7 (3)	C5—C6—C13—C15	136.9 (2)
C21—C22—C23—C24	179.1 (2)	C22—C21—N3—C17	−165.29 (17)
C22—C23—C24—C25	−0.4 (3)	C28—C21—N3—C17	83.59 (18)
C23—C24—C25—C26	1.3 (4)	C32—C21—N3—C17	−32.92 (19)
C24—C25—C26—C27	−1.0 (4)	C22—C21—N3—C20	−45.6 (3)
C25—C26—C27—C22	−0.2 (3)	C28—C21—N3—C20	−156.67 (19)
C25—C26—C27—N4	179.9 (2)	C32—C21—N3—C20	86.8 (2)
C23—C22—C27—C26	1.0 (3)	C18—C17—N3—C21	159.25 (19)
C21—C22—C27—C26	−178.8 (2)	C16—C17—N3—C21	36.4 (2)
C23—C22—C27—N4	−179.04 (18)	C18—C17—N3—C20	30.6 (2)
C21—C22—C27—N4	1.1 (2)	C16—C17—N3—C20	−92.22 (19)
N3—C21—C28—O3	−58.7 (3)	C19—C20—N3—C21	−160.7 (2)
C22—C21—C28—O3	−178.3 (2)	C19—C20—N3—C17	−40.8 (2)
C32—C21—C28—O3	55.8 (3)	O3—C28—N4—C27	178.9 (2)
N3—C21—C28—N4	120.64 (17)	C21—C28—N4—C27	−0.4 (2)
C22—C21—C28—N4	1.0 (2)	O3—C28—N4—C29	0.0 (3)
C32—C21—C28—N4	−124.93 (17)	C21—C28—N4—C29	−179.33 (19)
N4—C29—C30—C31	145.0 (4)	C26—C27—N4—C28	179.5 (2)
C15—C16—C32—C40	5.8 (2)	C22—C27—N4—C28	−0.4 (2)
C17—C16—C32—C40	−119.09 (17)	C26—C27—N4—C29	−1.7 (4)
C15—C16—C32—C33	−113.42 (18)	C22—C27—N4—C29	178.4 (2)
C17—C16—C32—C33	121.71 (17)	C30—C29—N4—C28	95.5 (3)
C15—C16—C32—C21	129.42 (17)	C30—C29—N4—C27	−83.2 (3)
C17—C16—C32—C21	4.56 (18)	C38—C39—O1—C40	159.71 (18)
N3—C21—C32—C40	140.09 (16)	C34—C39—O1—C40	−20.7 (3)
C22—C21—C32—C40	−90.1 (2)	C32—C40—O1—C39	57.4 (2)

### Hydrogen-bond geometry (Å, º)

Cg1 is the centroid of the C1–C6 ring.

**Table:**

*D*—H···*A*	*D*—H	H···*A*	*D*···*A*	*D*—H···*A*
O4*W*—H1*W*···N3^i^	0.91 (2)	2.02 (3)	2.892 (4)	161 (4)
O4*W*—H2*W*···O2^ii^	0.90 (1)	1.96 (1)	2.841 (3)	165 (3)
C40—H40*A*···*Cg*1^iii^	0.97	2.78	3.540 (3)	136

Symmetry codes: (i) −*x*+1/2, *y*+1/2, −*z*+1/2; (ii) *x*, *y*+1, *z*; (iii) −*x*+1, −*y*, −*z*.
